# Polypyrrole Decorated Flower-like and Rod-like ZnO Composites with Improved Microwave Absorption Performance

**DOI:** 10.3390/ma15093408

**Published:** 2022-05-09

**Authors:** Leilei Zhang, Yihua Lv, Xiaoyun Ye, Lian Ma, Song Chen, Yuping Wu, Qianting Wang

**Affiliations:** 1 School of Materials Science and Engineering, Fujian University of Technology, Fuzhou 350118, China; chinazll2022@163.com (L.Z.); lyh211202@163.com (Y.L.); mlajn@163.com (L.M.); gxchensong@126.com (S.C.); wyping1022@163.com (Y.W.); 2 Fujian Provincial Collaborative Innovation Center of Smart Green Mold, Fuzhou 350118, China; 3 School of Resources & Chemical Engineering, Sanming University, Sanming 365004, China

**Keywords:** ZnO, polypyrrole, composites, microwave absorption, dielectric loss

## Abstract

In this study, zinc oxide (ZnO)/polypyrrole (PPy) composites with flower- and rod-like structures were successfully fabricated by in situ polymerization and the hydrothermal method and used as microwave absorption (MWA) materials. The surface morphologies, crystal structures, and electromagnetic features of the as-prepared samples were measured by field-emission scanning electron microscopy (FE-SEM), energy dispersive spectroscopy (EDS), X-ray diffraction (XRD), and vector network analyzer (VNA). The results show that the conductive polymer PPy was successfully decorated on the surface of ZnO substrates. The MWA ability of flower- and rod-like ZnO/PPy composites is significantly enhanced after introduction of PPy. Rod-like ZnO/PPy composites exhibited superior MWA properties than those of flower-like ZnO/PPy. The former achieved a minimum reflection loss (*RL*_min_) of −59.7 dB at 15.8 GHz with a thickness of 2.7 mm, and the effective absorption bandwidth (EAB, *RL* < −10 dB) covered 6.4 GHz. PPy addition and stacked structure of rod-like ZnO/PPy composites can effectively improve the dielectric properties, form multiple reflections of incident electromagnetic waves, and generate an interfacial polarization effect, resulting in improved MWA properties of composite materials.

## 1. Introduction

In modern society, electromagnetic (EM) pollution has greatly increased with the continuous and rapid development of communication technology [1]. Therefore, microwave absorption (MWA) materials are being developed aiming at strong absorption and light weight. Over the last several decades, many conventional microwave absorbing materials have been studied, such as magnetic metal powders [2], ferrite [3,4], carbonyl iron [5], TiO_2_ [6], and ZnO [7]. These single-component EM materials generally have a narrow absorption band and low reflection loss. Therefore, to increase the MWA performance, new composite absorbing materials adjust the components, ratios, and structures of the composites by introducing conducting polymers [8,9,10,11,12,13], carbon materials [14,15,16], and other inorganic dielectric materials [17]. The combination of conductive polymers with semiconductor materials to improve the reflection loss of EM waves has attracted extensive attention from researchers. Polyaniline (PANI), Poly(3,4-ethylenedioxythiophene) (PEDOT), and polypyrrole (PPy) are commonly used conductive polymers. Among them, PPy has become a novel binding agent for MWA materials because of its positive performance, including tunable electrical conductance, good electron affinity, lower density, and good environmental stability [18]. The high dielectric loss of PPy can be used to prepare composites with strong MWA. Research shows that PPy addition can greatly improve the reflection loss of composite materials after compounding with carbon materials, leading to stronger EM absorbing capability [8,19]. It has been concluded that, under the action of alternating EM fields, polarization losses are generated in the composites, which, in turn, cause dielectric losses that increase the MWA performance.

ZnO is an n-type semiconductor material with a large band gap (3.37 ev) and high electron mobility and thermal conductivity [20]. As a functional material, ZnO is widely used in gas sensors, solar cells, catalysis, and other fields [21,22]. At the same time, ZnO has a relatively large dielectric constant and excellent dielectric loss and can be used as one of the components of MWA composites.

In this work, the ZnO/PPy composites comprising PPy-decorated flower- and rod-like ZnO substrates were fabricated by the hydrothermal method and in situ polymerization. The effect of ZnO substrates with different morphologies on the MWA properties of composites was then compared and discussed. The structure–activity relationship between the EM loss mechanism and the absorbing property of composites was also explored. This study offers guidance for the synthesis and application of conductive polymer/inorganic composites with different morphologies in the MWA field.

## 2. Materials and Methods

### 2.1. Materials

Pyrrole (C_4_H_5_N), zinc acetate dihydrate (Zn(CH_3_COO)_2_·2H_2_O), ferric chloride (FeCl_3_·6H_2_O), sodium p-toluenesulfonate(C_7_H_7_NaO_3_S), sodium hydroxide (NaOH), and potassium hydroxide (KOH) were purchased from Aladdin Biochemical Technology Co., Ltd. (Shanghai, China).

### 2.2. Preparation of ZnO Flower and ZnO Rod

A total of 25 mL NaOH (0.8 mol/L) aqueous solution and 25 mL Zn(CH_3_COO)_2_·2H_2_O aqueous solution (0.1 mol/L) were mixed and stirred for 15 min. The mixture was subsequently transferred to a 100 mL Teflon autoclave, and here the solvothermal environment was maintained at 110 °C for 8 h. After reaction, the product was collected by centrifugation. The product was rinsed with deionized water and ethanol repeatedly until the pH of supernatant was neutral. Finally, the flower-like ZnO was obtained under vacuum drying at 60 °C for 12 h.

Similarly, 25 mL KOH (2 mol/L) aqueous solution was slowly poured into 25 mL Zn(CH_3_COO)_2_·2H_2_O (0.8 mol/L) aqueous solution with continuous stirring to produce white precipitate. Then 30 mL H_2_O_2_ was injected with constant stirring for 1 h. The white precursor was separated by centrifugation with the following deionized water wash several times, and then vacuum drying was treated at 110 °C for 12 h. Subsequently, 0.3 g dried precursor was added to 40 mL KOH (0.075 mol/L) aqueous solution by fully stirring and then transferred to the hydrothermal reactor and maintained at 180 °C for 3 h. The gray precipitate was treated by the same separation and cleaning process as above. After vacuum drying at 65 °C for 12 h, rod-like ZnO was collected.

### 2.3. Preparation of Flower-like ZnO/PPy and Rod-like ZnO/PPy Composites

**Figure 1:**
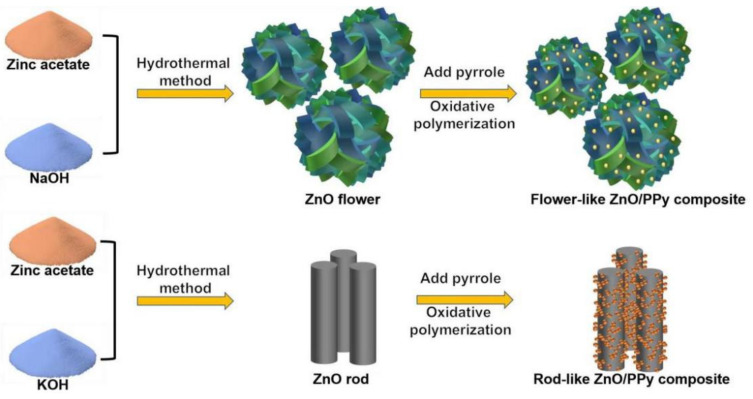
Schematic illustration of flower-like ZnO/PPy and rod-like ZnO/PPy composites.

A total of 0.3 g of the as-prepared ZnO powders and 2.0 g of sodium p-toluenesulfonate were added to 50 mL ethanol. The mixture was ultrasonic-dispersed for 30 min. A total of 0.8 mL pyrrole was dissolved in ethanol of 20 mL and fully stirred. The above two solutions were mixed for another 30 min with magnetic stirring. Then 20 mL FeCl_3_ solution (FeCl_3_/pyrrole = 1:1, molar ratio) was added to the mixture, and the polymerization reaction was maintained for 8 h. The precipitate was centrifuged and alternately washed with ethanol and deionized water and then vacuum dried for 12 h at 60 °C to obtain the composites. A schematic illustration of flower-like ZnO/PPy and rod-like ZnO/PPy composites is displayed in Figure 1.

### 2.4. Characterization

The surface morphologies of as-synthesized composites were analyzed by field emission scanning electron microscope (FESEM, NANONOVASEM 450, FEI). The composition and distribution of elements were observed with energy dispersive spectroscopy (EDS). The crystal structures of the samples were analyzed by powder X-ray diffraction (XRD, D8-ADVANCE, Bruker) under Cu Ka radiation (*λ* = 1.54056 Å) with a scanning rate of 4°/min in the 2θ range from 10 to 80°. The EM parameters of the samples were measured by a vector network analyzer (VNA, Agilent N5244A) in the frequency range of 2 to 18 GHz. The test samples were prepared as follows. The powder materials were mixed with paraffin with a mass ratio of 4:6 at 70 °C and pressed into a hollow cylindrical mold with an inner diameter of 3.00 mm and an outer diameter of 7.00 mm.

## 3. Results and Discussion

**Figure 2:**
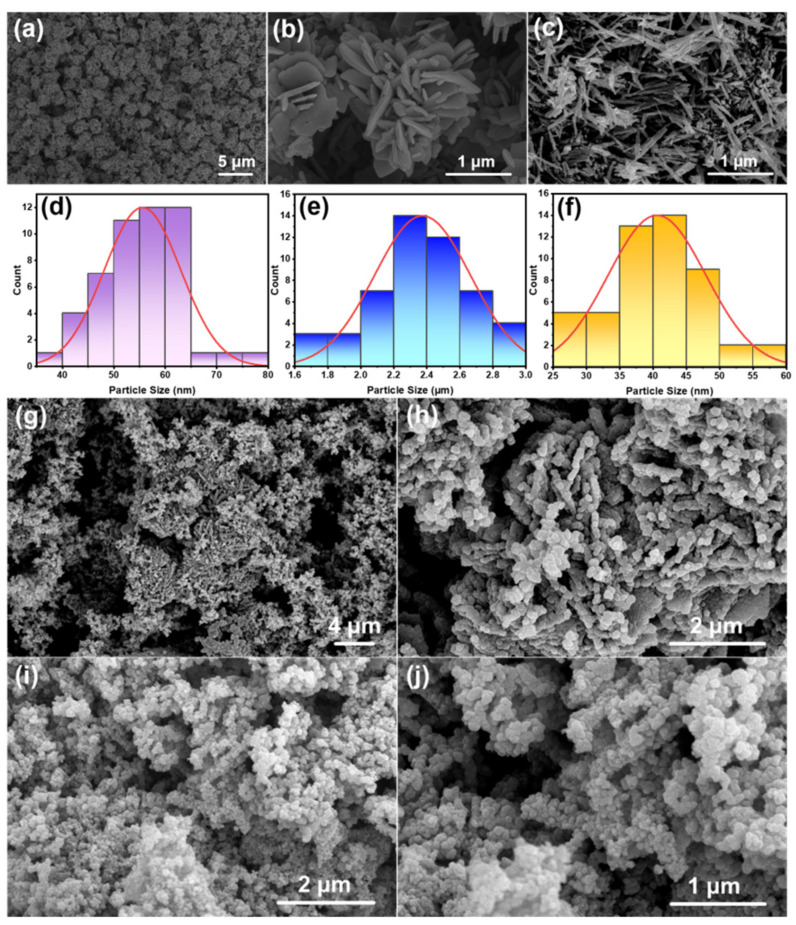
SEM images of the samples: (**a**,**b**) ZnO flowers, (**c**) ZnO rods, (**d**) diameter distribution chart of ZnO flowers, (**e**) petal thickness distribution chart of ZnO flower, (**f**) diameter distribution chart of ZnO rods, (**g**,**h**) flower-like ZnO/PPy composites, (**i**,**j**) rod-like ZnO/PPy composites.

SEM images of ZnO flowers, ZnO rods, flower-like ZnO/PPy composites, and rod-like ZnO/PPy composites, as well as a distribution chart of ZnO, are shown in Figure 2. It can be seen from Figure 2a,b,d,e that the smooth-faced petals of ZnO flowers are lamellar with thicknesses of approximately 55 nm. The mean diameter of ZnO flowers is approximately 2.4 μm. The length of ZnO rods varies from 300 to 800 nm with a mean diameter of about 40 nm in good dispersion (Figure 2c,f).

From the formation of the composites (Figure 2g–j), it can be observed that the surfaces of flower- and rod-like ZnO are decorated with PPy particles, resulting in obviously increased surface roughness. It is demonstrated that PPy was successfully polymerized on the surfaces of flower- and rod-like ZnO.

**Figure 3:**
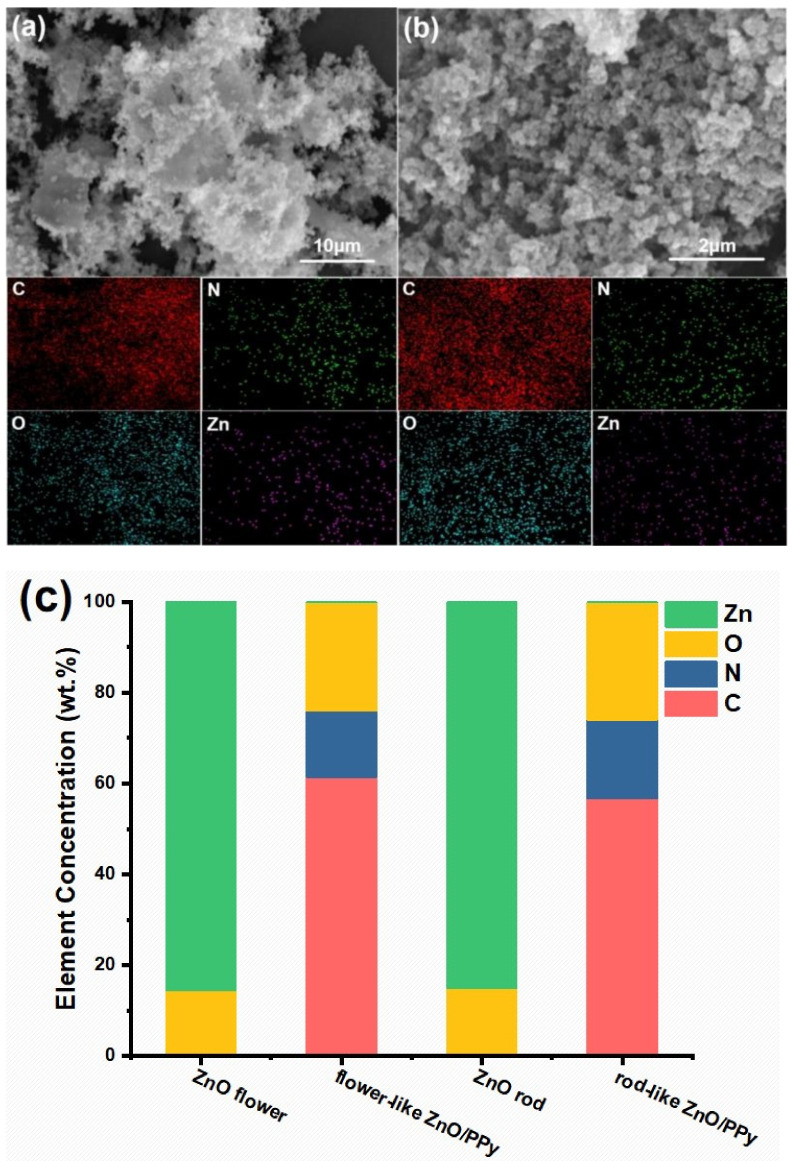
EDS mapping images of (**a**) flower-like ZnO/PPy composites and (**b**) rod-like ZnO/PPy composites; (**c**) quantitative analysis of ZnO flowers, ZnO rods, and corresponding ZnO/PPy composites.

To analyze the elemental composition of the samples, EDS mapping observation of flower- and rod-like ZnO/PPy composites and corresponding quantitative elemental analysis are represented in Figure 3. It can be seen from EDS mapping images of Figure 3a,b that four elements of C, O, N, and Zn are evenly distributed in flower- and rod-like ZnO/PPy composites. The corresponding quantitative elemental analysis (Figure 3c) shows that there are Zn and O elements in pure ZnO before the introduction of PPy. Additional two elements of C and N are detected after the formation of ZnO/PPy composites. The contents of three elements (C, N, and O) in flower- and rod-like ZnO/PPy composites are almost the same. Zn element is difficult to recognize because ZnO is wrapped by PPy, and thus the content signal of the Zn element can hardly be obtained in the composites. The element Zn is hardly detected probably because it is tightly wrapped by PPy.

**Figure 4:**
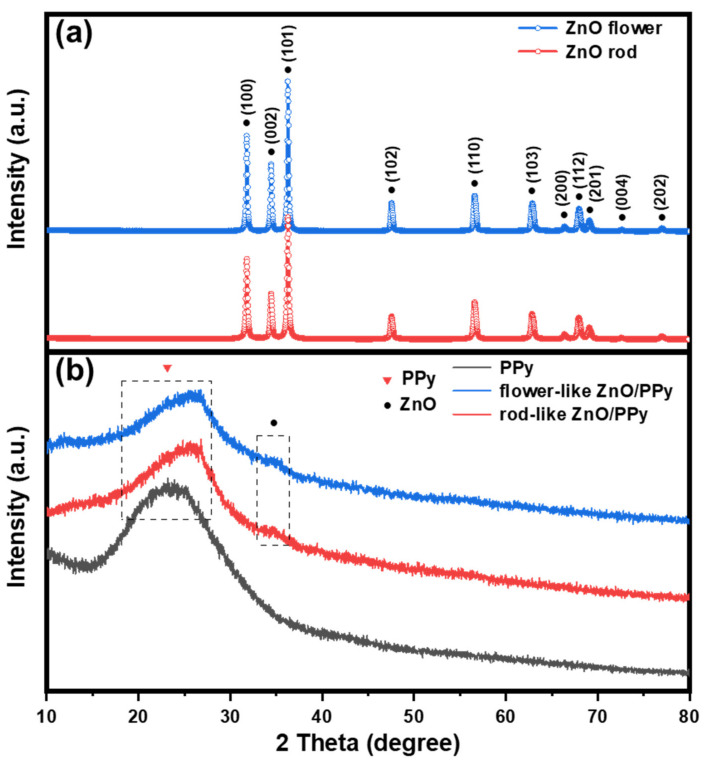
XRD patterns of (**a**) ZnO flower and ZnO rod; (**b**) PPy, flower-like ZnO/PPy, and rod-like ZnO/PPy composites.

Figure 4 shows the XRD curves of as-prepared ZnO flowers, ZnO rods, PPy, and ZnO/PPy composites with different flower- and rod-like morphologies plotted to investigate the crystallinity and phase composition of the samples. As shown, the characteristic diffraction peaks of ZnO flowers and ZnO rods located at 31.7°, 34.3°, 36.2°, 47.6°, 56.5°, 62.7°, 66.1°, 67.8°, 69.0°, 72.5°, and 77.8° correspond to the standard crystal planes of hexagonal wurtzite ZnO (JCPDS Card No. 75-1526). The clear sharp peaks indicate that the crystallinity of ZnO is good [23]. After introduction of PPy, a broad characteristic peak of amorphous PPy appears at 2θ = 24°, which originates from the disordered structure of the branch chains and crosslinks in molecular chain structures during pyrrole polymerization [19]. Meanwhile, the main diffraction peaks of ZnO are almost covered up, indicating that polymerization of PPy successfully occurs on the surfaces of ZnO and weakens the characteristic peaks of substrate ZnO.

**Figure 5:**
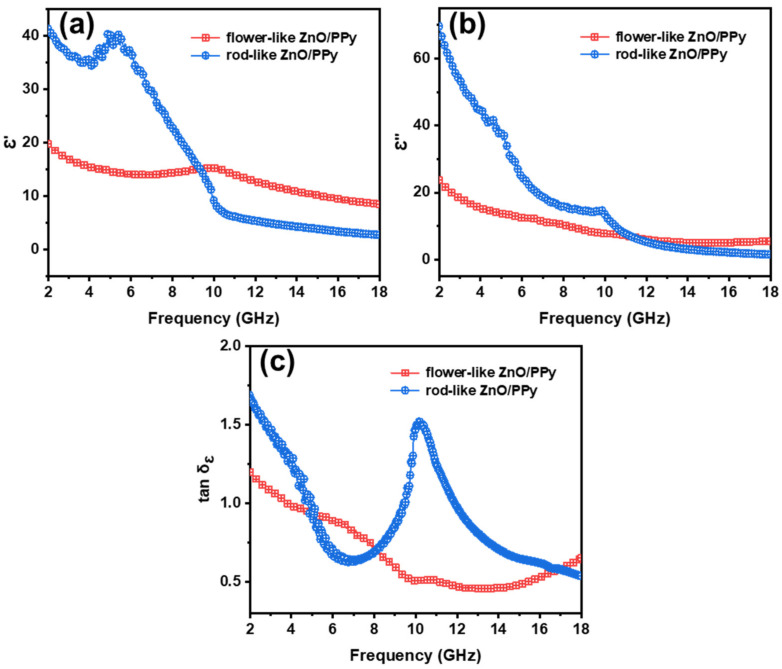
Frequency dependence of (**a**) real and (**b**) imaginary parts of complex permittivity; (**c**) dielectric loss tangent of flower-like ZnO/PPy and rod-like ZnO/PPy composites.

To compare the MWA properties of the composites, the real part ($\varepsilon^{\prime }$) of the permittivity, imaginary part ($\varepsilon^{\prime \prime }$) of the permittivity, and dielectric loss tangent ($\tan\delta_{\varepsilon }=\varepsilon^{\prime \prime }/\varepsilon^{\prime }$) of the samples, which represent the energy-storage ability, energy-dissipation ability, and dielectric loss capacity of the MWA materials, respectively, are shown in the range of 2–18 GHz in Figure 5. Compared to the flower-like ZnO/PPy composites, the $\varepsilon^{\prime }$ value of the rod-like ZnO/PPy composites is large when the frequency range is relatively low (Figure 5a). A significant resonance peak appears at 5.18 GHz, and then the $\varepsilon^{\prime }$ value decreases gradually for the rod-like ZnO/PPy composites, which means that it has better energy-storage ability. Furthermore, the $\varepsilon^{\prime \prime }$ value of the rod-like ZnO/PPy composites shows a resonance peak at 9.82 GHz (Figure 5b) that corresponds to the energy-dissipation ability of the samples. Figure 5c shows a remarkable relaxation peak of the rod-like ZnO/PPy composites near 10.2 GHz in the curve of $\tan\delta_{\varepsilon }$, which implies that ZnO/PPy composites with rod-like structure have a larger dielectric loss capacity and corresponding stronger reflection loss [24].

The MWA performance of the flower- and rod-like ZnO/PPy composites can be inspected by the reflection loss (*RL*) at different thicknesses in the range of 2–18 GHz. Transmission line theory assumes that the *RL* value is obtained by the electromagnetic parameters at a certain frequency and thickness according to the following formulas [25,26]:

(1) $$Z_{in}=Z_{0}\sqrt{\frac{\mu_{r}}{\varepsilon_{r}}}tanh\left[j\left(\frac{2\pi fd}{c}\right)\sqrt{\mu_{r}\varepsilon_{r}}\right]$$

(2) $$RL\left(dB\right)=20\;lg\;\left|\frac{Z_{in}-Z_{0}}{Z_{in}+Z_{0}}\right|\;$$

**Figure 6:**
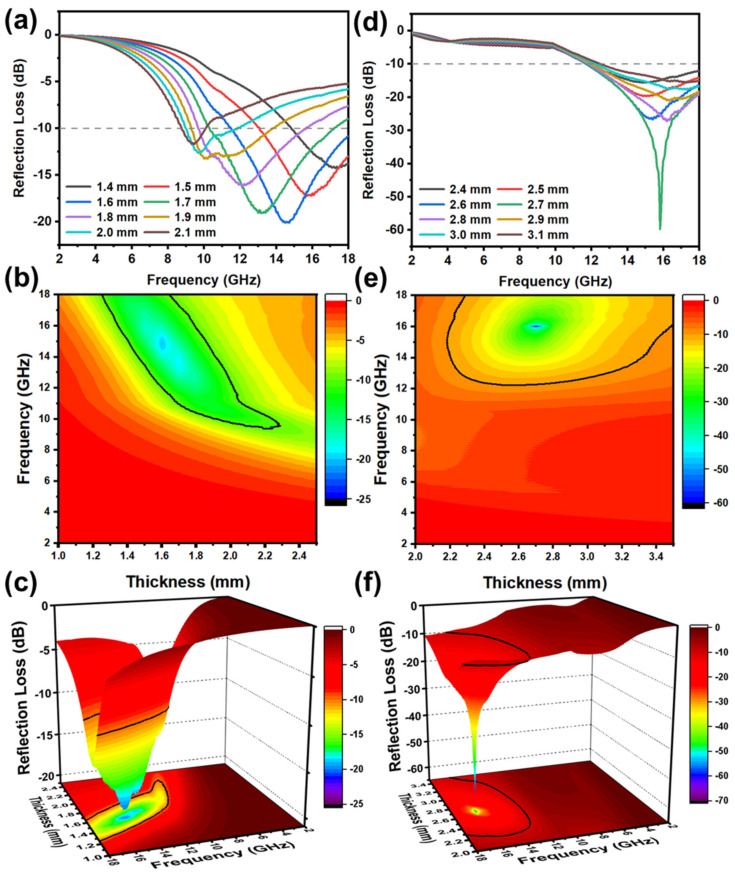
(**a**) The theoretical images, (**b**) 2D contours, and (**c**) 3D images of flower-like ZnO/PPy composites; (**d**) the theoretical images, (**e**) 2D contours, and (**f**) 3D images of rod-like ZnO/PPy composites.

Here, $Z_{in}$ represents the input impedance at the interface of free space and materials, $Z_{0}$ is the impedance of free space, *d* is the thickness of the absorber, and *μ_r_* and *ε_r_* are EM parameters of complex permeability and complex permittivity, respectively. Generally, the *RL* value of the materials (<−10 dB) means that the MWA reaches up to 90%. The calculated *RL* values of the theoretical, two-, and three-dimensional images of flower- and rod-like ZnO/PPy composites are shown in Figure 6. As can be seen from the figure, all the *RL* values of ZnO/PPy composites with different structures are less than −10 dB, indicating relatively good MWA capacity. In particular, the *RL*_min_ value of flower-like ZnO/PPy composites reaches −20.1 dB at 14.5 GHz, and the effective absorption bandwidth (EAB, <−10 dB) is 6.6 GHz when the thickness is 1.6 mm. For rod-like ZnO/PPy composites, *RL*_min_ reaches −59.7 dB at 15.8 GHz with an EAB of 6.4 GHz at a thickness of 2.7 mm. By comparing the above two ZnO/PPy composites, the MWA capacity of rod-like ZnO/PPy composites is found to be stronger than that of the flower-like composite structure. The MWA properties of the PPy-related and ZnO-related materials are listed in Table 1. By comparison, it is obvious that the rod-like ZnO/PPy composites exhibit strong microwave attenuation capacity, broad absorption bandwidth, and thin thickness. The rod-shaped composites with functional components have potential development in the field of microwave absorption.

It can be concluded from the above analysis that the MWA of ZnO/PPy composites can be expressed by dielectric loss. The process can be explained by Debye relaxation theory, as the following formula [32], which expresses the relationship between $\varepsilon^{\prime }$ and $\varepsilon^{\prime \prime }$.

(3) $${\left(\varepsilon^{\prime }-\frac{\varepsilon_{s}+\varepsilon_{\infty }}{2}\right)}^{2}+{\left(\varepsilon^{\prime \prime }\right)}^{2}={\left(\frac{\varepsilon_{s}-\varepsilon_{\infty }}{2}\right)}^{2}$$

where $\varepsilon_{s}$ and $\varepsilon_{\infty }$ represent the static and high-frequency permittivity, respectively. By plotting $\varepsilon^{\prime }$ and $\varepsilon^{\prime \prime }$, a Cole–Cole semicircle can be drawn out that exhibits the Debye relaxation process and that is related to the semicircle radius and number, as well as to the position of the frequency.

**Figure 7:**
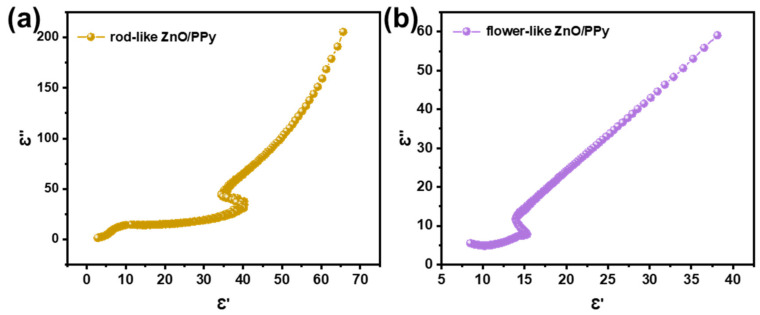
Cole–Cole semicircles of the composites: (**a**) flower-like ZnO/PPy, (**b**) rod-like ZnO/PPy.

To analyze the dielectric loss mechanism of flower- and rod-like ZnO/PPy composites, corresponding Cole–Cole semicircles are presented in Figure 7. The semicircle observed in each plot manifests that the relaxation process occurs in the composites [33], which is concerned with the interface polarization between ZnO and PPy. In addition, the semicircular radius of the rod-like ZnO/PPy composites is significantly larger than that of the flower-like ZnO/PPy composites, indicating a higher degree of polarization relaxation and interface polarization. The straight line that can be observed in the tail of the Cole–Cole curve illustrates dielectric loss dominated in the ZnO/PPy composites [34].

Transmission theory also explains that the attenuation constant (α) of the incident EM wave can further be used to estimate the microwave attenuation capability of the materials. The relationship between α and EM parameters ($\varepsilon^{\prime }$, $\varepsilon^{\prime \prime }$, $\mu^{\prime }$, and $\mu^{\prime \prime }$) is expressed as follows [35]:

(4) $$\alpha =\frac{\sqrt{2}\pi f}{c}\times \sqrt{\left(\mu^{\prime \prime }\varepsilon^{\prime \prime }-\mu^{\prime }\varepsilon^{\prime }\right)+\sqrt{{\left(\mu^{\prime \prime }\varepsilon^{\prime \prime }-\mu^{\prime }\varepsilon^{\prime }\right)}^{2}+{\left(\mu^{\prime \prime }\varepsilon^{\prime }-\mu^{\prime }\varepsilon^{\prime \prime }\right)}^{2}}}$$

**Figure 8:**
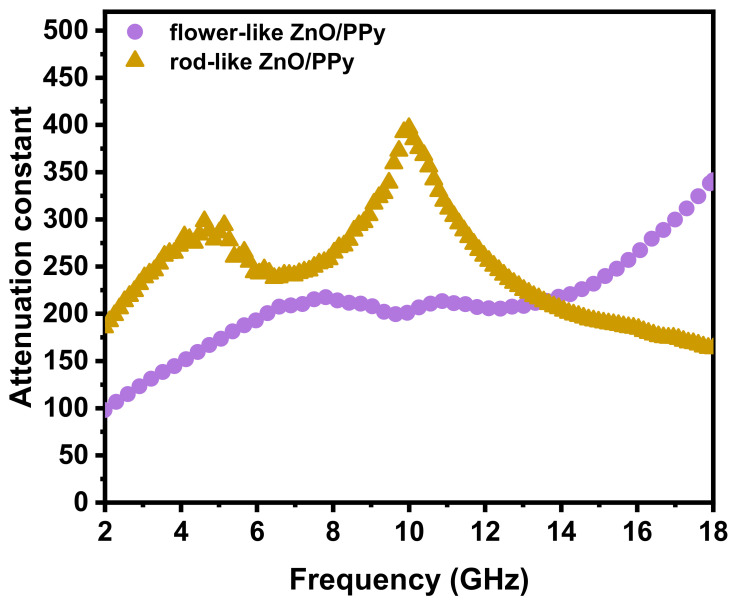
Microwave attenuation constant (α) of flower-like and rod-like ZnO/PPy composites.

The α values of flower- and rod-like ZnO/PPy composites are shown in Figure 8. Two peaks appear in the curve of the attenuation constant for rod-like ZnO/PPy composites, corresponding to high attenuation constant values of 298 and 396 at 4.6 and 9.9 GHz, respectively. Moreover, the α values of rod-like ZnO/PPy are higher than those of flower-like ZnO/PPy, indicating that the former has stronger attenuation ability of EM waves and better absorption performance. These results are consistent with previously presented reflection attenuation data.

**Figure 9:**
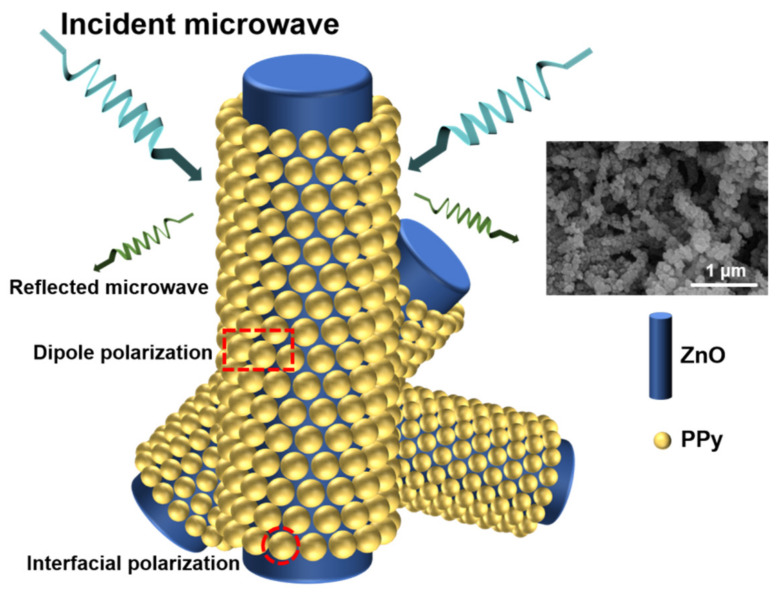
Microwave absorption mechanism of ZnO/PPy composites.

The MWA mechanism of ZnO/PPy composites is illustrated in Figure 9. The addition of conductive polymer PPy changes the dielectric constant due to a discrepancy in conductivity between PPy and ZnO, resulting in the uneven distribution of electrons in the composites. The charges accumulate at the interface between PPy and ZnO and generate interface polarization. Moreover, EM waves interact with charged particles from PPy to convert EM waves into heat energy [19]. At the same time, the rod-like ZnO/PPy at nanometer scale can be stacked with each other to form more gaps and effective channels, which can not only make the EM waves in the material to complete multiple reflections and scattering but can also enhance the interface dipole polarization to increase the dielectric relaxation, leading to the EM energy attenuation [36].

## 4. Conclusions

ZnO/PPy composites with flower- and rod-like structures were successfully fabricated by in situ polymerization and the hydrothermal method. The introduction of PPy effectively improves the microwave attenuation capacity of the ZnO/PPy composites. The rod-like ZnO/PPy composites show superior MWA performance than those of the flower-like ZnO/PPy composites. The rod-like ZnO/PPy composites show an *RL*_min_ value of −59.7 dB and EAB of 6.4 GHz at a thickness of 2.7 mm. The excellent MWA performance of the composites was mainly ascribed to the microstructure, dielectric properties of the components, and multiple interfacial polarization between ZnO and PPy. The study for the design of conductive polymer/inorganic composite materials with different morphologies provides new guidance in the MWA field. The designed conductive polymer/inorganic composite materials have control over the morphology, particle size distribution, and composition by a hydrothermal/solvothermal method, which might be a promising synthesis technique in the fabrication of materials with superior MWA properties.

## Figures and Tables

**Table 1:** MWA properties of related absorbers.

Absorber	*RL*_min_ (dB)	Optimum Thickness (mm)	Bandwidth (GHz) *RL* < −10 dB	References
ZnO nanoparticles	−37.7	2.1	3.1	[7]
Ti_3_C_2_T_X_@PPy	−49.5	3.6	5.1	[19]
ZnO/MoS_2_	−35.8	2.5	10.2	[24]
PPy nanorods	−45	3.1	5.6	[27]
ZnO nws/RGO foam/PDMS	−27.8	4.8	4.2	[28]
Co/PPy	−33	2.0	4.7	[29]
CoFe_2_O_4_@PPy	−43.8	3.0	5.7	[30]
CoZn/C@MoS_2_@PPy	−49.1	1.5	4.5	[31]
Rod-like ZnO/PPy	−59.7	2.7	6.4	This work

## Data Availability

Not applicable.
